# Optimal Growth Conditions for Forming *c*-Axis (002) Aluminum Nitride Thin Films as a Buffer Layer for Hexagonal Gallium Nitride Thin Films Produced with In Situ Continual Radio Frequency Sputtering

**DOI:** 10.3390/mi13091546

**Published:** 2022-09-17

**Authors:** Wei-Sheng Liu, Balaji Gururajan, Sui-Hua Wu, Li-Cheng Huang, Chung-Kai Chi, Yu-Lun Jiang, Hsing-Chun Kuo

**Affiliations:** 1 Department of Electrical Engineering, Yuan Ze University, Chung-Li Dist., Taoyuan 32003, Taiwan; 2 Department of Nursing, Division of Basic Medical Sciences, Chang Gung University of Science and Technology, Chiayi 61363, Taiwan; 3 Research Fellow, Chang Gung Memorial Hospital, Chiayi 61363, Taiwan; 4 Research Center for Food and Cosmetic Safety, College of Human Ecology, Chang Gung University of Science and Technology, Taoyuan 33302, Taiwan; 5 Chronic Diseases and Health Promotion Research Center, Chang Gung University of Science and Technology, Chiayi 61363, Taiwan

**Keywords:** aluminum nitride, reactive sputtering, X-ray diffraction, X-ray photoelectron spectroscopy, hardness measurement

## Abstract

Aluminum nitride (AlN) thin-film materials possess a wide energy gap; thus, they are suitable for use in various optoelectronic devices. In this study, AlN thin films were deposited using radio frequency magnetron sputtering with an Al sputtering target and N_2_ as the reactive gas. The N_2_ working gas flow rate was varied among 20, 30, and 40 sccm to optimize the AlN thin film growth. The optimal AlN thin film was produced with 40 sccm N_2_ flow at 500 W under 100% N_2_ gas and at 600 °C. The films were studied using X-ray diffraction and had (002) phase orientation. X-ray photoelectron spectroscopy was used to determine the atomic content of the optimal film to be Al, 32%; N, 52%; and O, 12% at 100 nm beneath the surface of the thin film. The film was also investigated through atomic force microscopy and had a root mean square roughness of 2.57 nm and a hardness of 76.21 GPa. Finally, in situ continual sputtering was used to produce a gallium nitride (GaN) layer on Si with the AlN thin film as a buffer layer. The AlN thin films investigated in this study have excellent material properties, and the proposed process could be a less expensive method of growing high-quality GaN thin films for various applications in GaN-based power transistors and Si integrated circuits.

## 1. Introduction

Aluminum nitride (AlN) thin films have excellent material properties. They can be used in various advanced optoelectronic devices and can be integrated with high-power gallium nitride (GaN) transistors in Si-based integrated circuits. The superior material properties of AlN films include a wide energy bandgap of approximately 6.2 eV, high thermal conductivity (up to 320 W/mK), high thin-film resistivity (10^11^–10^13^ Ω cm), high breakdown field (5 MV/cm), good thermal stability, and a low thermal expansion coefficient. In addition, AlN films are promising candidates for applications in piezoelectric energy harvesting, thin-film bulk acoustic resonators, optoelectronics, short wavelength-light sources and detectors, sensors, and actuators [1,2,3,4,5,6].

An AlN thin-film layer can be deposited using various techniques, such as metal organic chemical vapor deposition (MOCVD) [7], molecular beam epitaxy (MBE) [8], dual-ion beam sputtering [9], and pulsed laser ablation [10]. The deposition parameters can be varied to study their effects on the material quality of AlN films. However, the techniques listed are expensive and require ultra-high vacuum and high growth temperatures.

Direct current (DC) and radio frequency (RF) sputtering [11] are inexpensive and can be performed at low temperatures; thus, they are suitable for fabricating microelectromechanical systems (MEMS) devices. By using multiple chambers, sequential in-line thin-film sputtering deposition for optoelectronic devices can be performed without breaking the vacuum, thereby decreasing oxidation and preventing the degradation of the device’s characteristics, which is consequently cost-effective and increases the production capacity of AlN-based optoelectronic devices. To grow high-quality (002) *c*-axis-oriented AlN thin films with smooth thin-film surfaces through reactive sputtering, supplying the kinetic energy by optimizing the sputtering parameters for the adatoms on the surface of the growing thin films is essential. AlN films with (002) *c*-axis orientation have a large electromechanical coupling coefficient and good piezoelectricity; these characteristics are useful in developing high-frequency devices, such as surface and bulk acoustic wave devices, used in mobile phones and other common communication devices [12].

Group III-nitride compound semiconductor materials have gradually attracted the attention of researchers for use in high-power electronic devices because of their excellent material properties, such as their high material energy bandgap, thermal and mechanical stability, superior electron saturation and peak velocities, high breakdown voltage and thermal conductivity, and excellent radiation hardness. In addition, group III-nitride compound semiconductor materials have wide and tunable material direct energy bandgaps of 6.2, 3.4, and 0.7–1.9 eV for AlN, GaN, and indium nitride (InN), respectively. InN and AlN can be alloyed with GaN to form ternary or quaternary compound semiconductor alloys, enabling the modulation of the energy gap of the design alloy material and the emission wavelength to meet the specific requirements of the optoelectronic device. AlN is often employed as an epitaxial buffer layer for the crystal growth of GaN films in GaN-based optoelectronic devices for applications in high-frequency microwave transistors or high-power electric vehicles because AlN and GaN films have similar thermal expansion coefficients, crystal structures, and lattice constants. Thus, the lattice mismatch degree between sapphire/Si substrates and GaN films is small, improving the performance of GaN-based optoelectronic devices [13,14,15].

Several key papers on the preparation of AlN thin films have been published. Iborra et al. [16] deposited highly *c*-axis-oriented AlN thin films by using RF reactive sputtering for MEMS applications through the modulation and optimization of the thin-film sputtering deposition parameters. Singh et al. [17] deposited AlN thin films over Mo/SiO2/Si substrate and varied the N_2_ flow rate to study its effect on the material qualities of AlN films. The films were characterized using techniques such as X-ray diffraction (XRD) for full width at half maximum (FWHM) studies, atomic force microscopy (AFM), and scanning electron microscopy. Finally, they fabricated a capacitor with a Mo–AlN–Mo structure and studied its capacitance; it had a dielectric constant of 8.89 with a capacitance value of 42 pF measured through the top electrode area of 400 × 400 μm^2^. Zhang et al. [18] deposited (002) *c*-axis AlN films over Si (111) and Si (100) substrates. In addition, they conducted extensive research on the modulation of the sputtering power from 200 to 500 W and discovered that the FWHM of the XRD diffraction could be decreased from 0.43° to 0.32°, indicating that the AlN film improved crystal quality. Medjani et al. [19] deposited AlN films on Si (100) by using RF magnetron sputtering in a mixed argon and nitrogen atmosphere with a negative substrate bias of −100 V at varying thin-film deposition temperatures of up to 800 °C. The results revealed that the deposition of AlN thin films at lower growth temperatures and with adequate substrate bias facilitated AlN film growth with the formation of the (002) plane parallel to the substrate surface. Kar et al. [20] studied the morphology and orientation of AlN films deposited over a *p*-type Si substrate in terms of the changes in sputtering pressure. As the sputtering pressure increased, the quality of the AlN (002) crystal improved, and a phase orientation change to (100) was observed at a growth pressure of 6 mTorr. As the sputtering pressure increased from 4.5 to 6 mTorr, the surface roughness increased from 1.56 to 3.24 nm, and the grain size decreased from 114 to 80 nm. Guo et al. [21] discussed the effects of varying RF power during the deposition of AlN films over a sapphire substrate and reported that the deposition rate and surface roughness of the AlN film increased and decreased, respectively, as the power was increased. In addition, they observed that the AlN film had a high optical transmission in both the visible and ultraviolet ranges. Consequently, they determined that increasing the RF sputtering power results in more crystal defects in AlN films.

A few studies have been conducted on the properties of AlN films deposited using RF sputtering. The relationship of sputtering growth pressure with the crystal quality and orientation of AlN films is clear. A decrease in pressure could cause the transition of AlN films from (100) to (002) phases and a decrease in the FWHM of the XRD rocking curve. Therefore, an in-depth study is required to optimize all the parameters required to obtain (002) AlN with superior crystal thin-film quality for use as a buffer layer to facilitate the crystal growth of GaN thin films. In this study, we deposited AlN thin films over Si substrate by using RF sputtering.

Several studies have been conducted on the epitaxial growth of GaN thin films over an AlN buffer layer for various applications. Studies have improved the understanding of the fabrication of AlGaN/GaN high–electron mobility transistors (HEMT). Improved devices, such as the HEMT, have been created because of the use of higher-performance methods of depositing GaN over AlN. Almost all research groups have fabricated the HEMT devices by using MBE or MOCVD for the subsequent deposition of layers [22,23]. Understanding the behavior of GaN and AlN layers on the Si substrate enables investigation into improving the properties through alternate vacuum deposition methods. Direct epitaxial growth of GaN on Si is challenging, primarily because of the high reactivity of the Si surface with N_2_ and group III species, as well as the high lattice mismatch between GaN and Si and the thermal expansion coefficient of GaN [24]. By using AlN as a buffer layer beneath GaN, the nucleation density of the islands can be increased. Therefore, studying the various aspects of deposition and the properties of AlN as a buffer layer over Si is necessary for GaN-based sputtering applications.

We explored the effects of deposition parameters, namely sputtering power, substrate temperature, and nitrogen flow ratio, on the crystallization characteristics of AlN thin films. We demonstrated the excellent crystallization characteristics of optimized (002) AlN films. In addition, we studied the deposition of GaN on Si (111) with AlN as a buffer layer. All the depositions were performed using RF sputtering without breaking vacuum.

## 2. Materials and Methods

The AlN thin films were deposited on *p*-type Si (111) substrates by using an RF sputtering system. The Si substrates were thoroughly cleaned using acetone, isopropyl alcohol, and distilled water, and the removal of native oxide was performed using buffered oxide etch. A 3″ Al target (99.999%) was used for the AlN thin-film sputtering deposition, with a working distance of 7 cm between the substrate and Al target. Before thin-film sputtering deposition, the growth chamber was evacuated to a base pressure of 4 × 10^−6^ Torr. During sputtering deposition, the working pressure was maintained at 3 mTorr, and the substrate holder was rotated at 10 RPM. Prior to thin-film sputtering deposition, pre-sputtering was performed for 20 min by using argon gas to clean the Al target, and pure nitrogen gas was then introduced to deposit 450 nm-thick AlN thin films.

In this study, four sets of AlN samples were prepared with different sputtering deposition conditions. The first set of AlN samples was sputter-deposited with a power of 300, 400, and 500 W to study the effect of sputtering power on the crystal quality of the AlN thin film; the substrate temperature was maintained at 600 ℃, and the total N flow rate was maintained at 40 sccm. Subsequently, the sputtering power was maintained at 500 W, and the nitrogen flow ratio was set to 50%, 75%, or 100% for the second set of AlN samples. The effect of the thin-film deposition temperature on the AlN crystal quality was studied in the third set of samples; elevated substrate temperatures between 400 and 800 °C were investigated. Finally, the total nitrogen flow was set between 20 and 40 sccm for the fourth set of AlN samples. By optimizing the sputtering parameters, (002)-orientated AlN thin films with superior crystal quality grown on Si (111) substrates were obtained.

Subsequently, GaN thin films were deposited on the AlN buffer layer on Si (111) substrate as Si/AlN/GaN. The depositions were performed in the same RF sputtering chamber without breaking the vacuum. After the AlN thin films were deposited over Si (111) substrate, 45 g of Ga ingots (8N) were melted into a custom holder that was used as the sputtering target. N_2_ gas was used as the reactive gas for the deposition of the GaN layer. The sputtering power was maintained at 100 W, the substrate temperature was 600 ℃, and pre-sputtering was performed for 5 min before the shutter was opened for Ga deposition. The N flow was maintained at 40 sccm. Finally, the Si/AlN/GaN stack was deposited using RF sputtering.

To measure and analyze the material quality of the AlN thin films, identification of crystalline phases and related crystal quality studies were performed using XRD (Bruker D8, Karlsruhe, Germany). A uTek Nanoview 1000 was used for AFM to study the morphology and surface roughness of the AlN thin films. The chemical composition of the AlN thin films was investigated using a PHI 5000 Versa Probe Ⅲ X-ray Photoelectron Spectroscopy unit (ULVAC-PHI). Nanoindentation (thin-film hardness) was studied using Hysitron TI 980 TriboIndenter with the maximum force maintained at 1 nN.

## 3. Results

### 3.1. XRD

#### 3.1.1. Change in Sputtering Power and Nitrogen Flow Ratios

**Figure 1:**
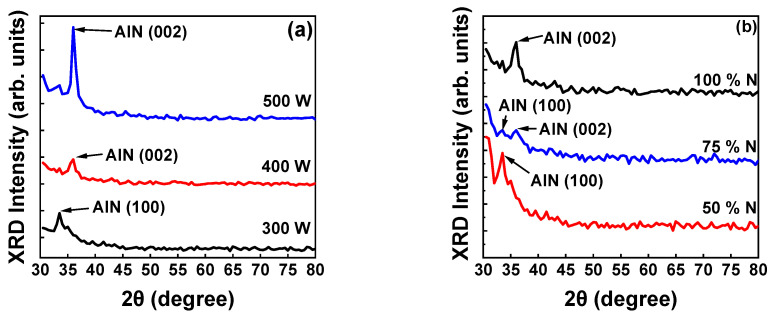
(**a**) XRD pattern of AlN (002) thin films deposited using sputtering powers of 300, 400, and 500 W. (**b**) XRD pattern of AlN (002) thin films deposited with nitrogen flow ratios of 50%, 75%, and 100% and a sputtering power of 500 W.

The XRD patterns for the first set of AlN thin films deposited with sputtering powers of 300, 400, and 500 W revealed a polycrystalline structure (Figure 1a) and were analyzed with reference to the AlN JCPDS card No. 25-1133.

Figure 1a reveals that increasing the sputtering power from 300 to 400 W caused the crystallographic phase of the AlN film to transition from the (100) to the (002) crystallographic orientation because of the high kinetic energy of the surface adatoms, resulting in the sputtering growth of (002)-orientated AlN film [24,25]. As the sputtering power was further increased to 500 W, the surface atoms of the AlN film received more energy, resulting in preferential crystal growth in the (002) direction; the intensity of the AlN sample deposited at 500 W had a three-fold increase in the intensity when compared to the other two samples as seen from Figure 1a. Studies have reported that sputtering growth parameters are key in the formation of the (100) phase and the phase transition to the (002) phase [26,27]. The XRD results for the first set of AlN films reveal that an increase in sputtering power considerably increases the growth of AlN thin films with a (002) orientation; therefore, 500 W was used for AlN thin film sputtering growth in subsequent experiments. Figure 1b presents the X-ray diffraction patterns of AlN films sputter-deposited using N_2_ flow ratios of 50%, 75%, and 100% at 500 W. The X-ray diffraction pattern in Figure 1b reveals that as the N_2_ flow ratio increases, the crystallographic orientation of the AlN film transitions from an AlN (100)- to (002)-preferential crystallographic orientation. Thus, the crystalline orientation of AlN films is affected by not only the sputtering power but also the N_2_ and Ar working gas ratio.

AlN (002) is considered the most favorable phase for AlN thin films for numerous reasons. In periodic bond chain (PBC) theory, a set of uninterrupted chains of strong bonds form a crystal lattice. The lattice is defined by its attachment energy E(att); that is, the amount of energy released per mole as a new layer is deposited on the crystal lattice. The growth rate of a thin film depends on both the sputtering power and N_2_ flow ratios. The XRD pattern result can be corroborated theoretically by considering the formation of Al–N bonds. Wurtzite AlN is typically formed in the (001), (100), and (101) phases. These are the slow-growing phases (or the flat phases) from the AlN PBC. (001) is considered a close-packed crystal. In general, as the N_2_ flow ratio increases, the crystals already formed with phase (001) receive more N_2_ atoms and tend to form new Al–N bonds. Bombardment by ions in the plasma results in the destruction of crystals with phases (101), (001), and (100) because the atoms adsorbed on the surface of the loosely packed crystals cause a transition to the (002) phase. The E(att) of N atoms is high for a 100% N_2_ flow ratio, and when N atoms reach a thin-film surface with the (001) phase, the formation of the (002) phase is enhanced. If N atoms are more numerous, the (002) growth rate is faster. This theoretical explanation is supported by the XRD plot; as both the sputtering power and N_2_ flow ratio increase, the (002) phase is increasingly preferred over the (100) phase [28].

Figure 1a,b clearly indicates the crystal orientation transitions from (100) to (002) when the sputtering power increased, and the N_2_ flow ratio was 100%. At a sputtering power of 500 W, sufficient kinetic energy was provided to form the (002) orientation; simultaneously, the N_2_ flow ratio was maintained at 100% to decrease the deposition rate, stabilizing the (002) orientation [29]. The sputtering power and N_2_ flow ratio were changed to identify the effects of increased sputtering power and N_2_ flow ratio for the deposition of AlN thin films with (002) crystal orientation; the optimal sputtering power and N_2_ flow ratio were 500 W and 100%, respectively. To produce AlN films with superior crystal quality, the optimal parameters of 500 W sputtering power and 100% N_2_ flow ratio were maintained in subsequent investigations.

#### 3.1.2. Effects of Thin-Film Growth Temperature and N_2_ Flow Rate

**Figure 2:**
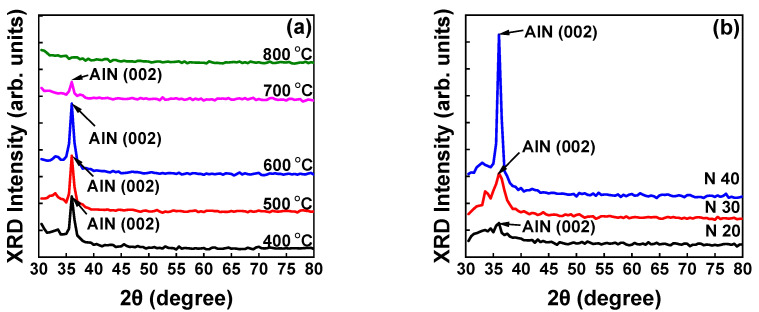
(**a**) XRD pattern of AlN (002) deposited at temperatures ranging from 400 to 800 °C, (**b**) XRD pattern of AlN (002) deposited by varying the nitrogen flow rate from 20 to 40 sccm.

Figure 2a presents the XRD patterns for AlN thin films on Si (111) substrates at various growth temperatures ranging from 400 to 800 °C. The predominant XRD peak was observed at 36°, corresponding to the AlN (002) phase. The XRD intensity of the AlN (002) peak increased as the deposition temperature increased from 400 to 600 °C but decreased at 700 °C and disappeared at 800 °C, indicating that at high temperatures, the AlN film deteriorated and had an amorphous structure. Thus, the XRD results reveal that 600 °C is the optimum growth temperature for AlN thin films.

At higher substrate temperatures, the energy of the thin-film surface adatoms increased, resulting in an increase in adatom mobility and surface diffusion length. The increase in adatom mobility and surface diffusion length facilitated the formation of the (002) orientation. As the temperature increased further to 700 °C, the desorption rate at the substrate surface further increased and decreased the formation of the (002) orientation, increasing the FWHM of (002) orientation [30]. The values of FWHM were calculated to be approximately 0.763°, 0.724°, 0.716°, and 0.914° for the AlN films grown at 400, 500, 600, and 700 °C, respectively. Further optimization, such as by varying the N_2_ flow, was performed at the optimal temperature of 600 °C.

Figure 2b presents the XRD pattern of AlN thin films deposited with N_2_ flow rates of 20, 30, and 40 sccm and sputtering power, N_2_ flow ratio, and growth temperature maintained at 500 W, 100%, and 600 °C, respectively. As the nitrogen flow rate increased, the chance of collision between N and Al atoms increased. Thus, the chemical bonding between N and Al also increased, and the formation of AlN films was consequently enhanced. As the nitrogen flow rate increased from 20 to 40 sccm, the XRD intensity also increased, indicating that the crystalline quality of the AlN thin film was improved.

### 3.2. X-ray Photoelectron Spectroscopy

**Figure 3:**
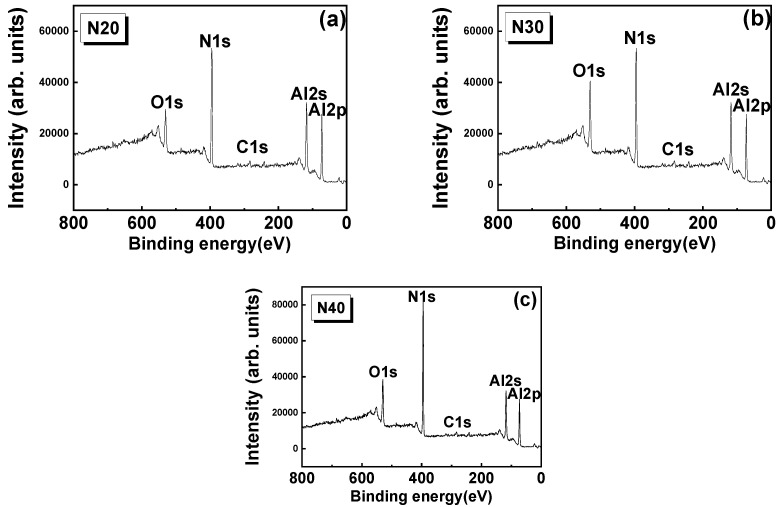
XPS survey spectrum of AlN thin films sputtering deposited using N_2_ flow rate of (**a**) 20, (**b**) 30, and (**c**) 40 sccm.

Figure 3 presents the X-ray photoelectron spectroscopy (XPS) survey spectrum for AlN thin films sputter-deposited at N_2_ flow rates of 20, 30, and 40 sccm. The survey spectrum reveals the presence of O 1s and C 1s at 285 and 532 eV, respectively. The presence of C and O was attributed to atmospheric contamination and surface oxidation, respectively. The AlN film tends to form several surface oxides and hydrides, as has been reported in the literature [31,32]. The O and C may form because of various sample environments and conditions. The presence of Al 2s and 2p and N 1s can also be observed in the spectra. Thus, the survey spectrum demonstrates the formation of AlN, and the intensity of the N peak indicates that as the N_2_ flow rate increased, the concentration of N_2_ (i.e., the XPS intensity) increased.

**Figure 4:**
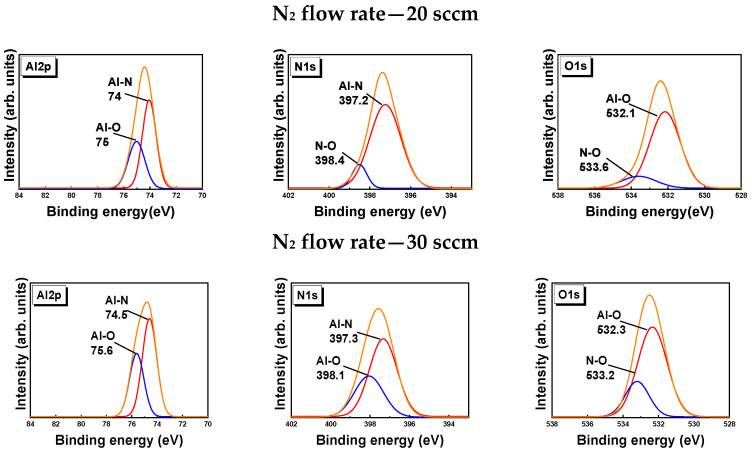

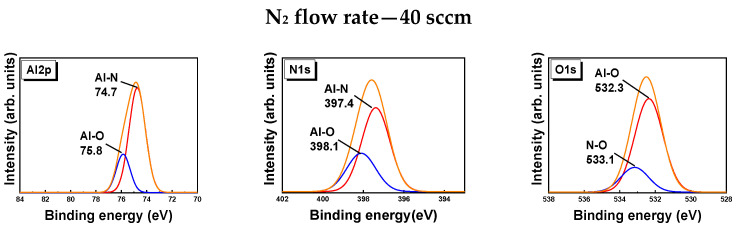
XPS core level spectra for Al, N, and O for various N_2_ flow rates.

Figure 4 presents the core level spectra of Al 2p, O 1s, and N 1s for the AlN thin films sputtered with N_2_ flow rates of 20, 30, and 40 sccm. The Al 2p core level peak reveals the nature of Al–N bonding in the film. Studies [32,33] have reported that the formation of the Al-N bond occurs at approximately 74.5–75 eV [33,34,35]. Gaussian fitting of the core level scans of our samples revealed Al 2p peaks at 74, 74.5, and 74.7 eV for the AlN films grown with N_2_ flow rates of 20, 30, and 40 sccm, respectively. The core level plots thus indicate the formation of AlN. The Al–O bond formed at 75.6 eV with respect to Al 2p, as revealed by the deconvoluted core level plot of Al 2p at 30 sccm. The Al–O bonds formed at 75, 75.6, and 75. 8 eV for N_2_ flow rates of 20, 30, and 40 sccm, respectively. Generally, AlN has more affinity to O, and O tends to be accommodated in the AlN structure, as reported in several studies [31,32]. The presence of O_2_ in the sample was primarily attributed to surface oxidation.

Core level N 1s peaks were studied to understand the formation of AlN. The results reveal that the core level peak of N 1s was centered around 397 eV; the peaks around 397 eV were deconvoluted to investigate Al–N–O bonding. The centered peak is consistent with results in the literature [32,33]. The deconvoluted data reveal the formation of Al–N at 397.2, 397.3, and 397. 4 eV for N_2_ flow rates of 20, 30, and 40 sccm, respectively. A weak peak was observed at 398.1 eV for Al–O [34,35,36]. The weak peak at 398.1 eV may also be due to surface oxidation.

The core level plots of O 1s reveal that the area under the peak is large and indicates greater O_2_ content in the samples. Al_2_O_3_ forms at 531.1 eV with respect to O [37]. The core level plots reveal that the O_2_ core level peak is centered around 532 eV. However, as the area under the curve increased, more O_2_ was observed in the thin films, indicating the formation of more Al–O bonds in the films [38,39,40]. The binding energy values from the core level plots of the O 1s and N 1s peaks indicate no formation of any oxynitride bonds; thus, AlN formed with some O_2_ atoms on the surface of the AlN thin-film layers [41,42,43].

Table 1 presents various atomic percentages (at%) of Al, N, and O observed in the AlN thin films. The surface level values were calculated using the following relation, and the at% at depth were obtained directly from the Casa XPS software (Version–2.3.25; CASA Pvt Ltd., Devon, United Kingdom):

(1) $$at\%\;i=\frac{\frac{A_{i}}{F_{i}}}{\sum i\;\frac{A_{i}}{F_{i}}}$$

where *A_i_* represents the area of the *i*th element and *F_i_* is the relative sensitivity factor.

The percentage of N_2_ increased as the N_2_ flow rate increased. However, O_2_ content due to thin-film surface oxidation is unavoidable. The remaining at% comprises C that is present because of atmospheric contamination. The at% of Al, N, and O at the surface and at depths of 50 and 100 nm was also determined. As the N_2_ flow rate increased from 20 to 40 sccm, the at% of Al increased, indicating that the impurities present in the plasma during sputtering were reduced, leading to the formation of a superior AlN thin film. For the AlN thin films etched to 50 or 100 nm, the at% of Al and N increased, but that of O decreased, indicating a reduction in Al–O bonding and AlN thin films with increased purity. The presence of O on the surface was due to surface oxidation; however, the presence of O in the bulk must be investigated to achieve pure AlN thin films. The presence of O in the bulk may be due to impurities in the plasma, oxygen-related defect complexes, or oxygen point defects in AlN [44]. Oxygen affinity toward AlN is high, and thus, the formation of intermediate Al–O–N phases is inevitable. The intermediate Al–O–N phases were then transformed to AlN, Al_2_O_3_, or AlO_x_N_y_. V_N_ (N vacancies) formed during sputtering; O attempted to occupy the N vacancies, forming Al–O bonds. A physical parameter might have helped O occupy V_N_. For example, O may have been released from the sputtering chamber walls during the experiment because of the cooling systems and moisture content of the chamber. Studies [45,46] have suggested various reasons for O in experimental AlN films produced through sophisticated methods such as MBE, MOCVD, or ALD. The presence of O during sputtering is thus not unexpected, particularly because the pressures in sputtering are significantly lower than those in other deposition techniques.

**Figure 5:**
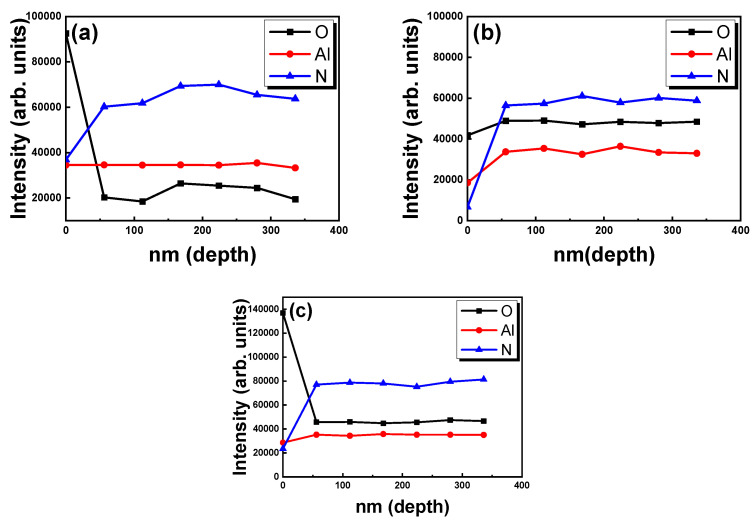
Depth profile analysis for AlN thin film samples with N_2_ flow rates of (**a**) 20, (**b**) 30, and (**c**) 40 sccm.

Figure 5 presents the depth analysis of the AlN thin films sputtering deposited at N_2_ flow rates of 20, 30, and 40 sccm. The surface O_2_ level of the AlN films can be attributed to oxidation. However, the bulk of the AlN increased N and decreased O content, indicating that sputter-grown AlN films have uniform Al–N bonding except for the surface level oxidation. Therefore, if the AlN film is used as a buffer layer for a subsequent GaN layer in the continual vacuum sputtering process, breakage of the vacuum of the sputtering system must be avoided to decrease the oxidation of the surface of the AlN film and prevent the degradation of GaN-based optoelectronic devices.

### 3.3. AFM

High-quality AlN films with flat surfaces are key to the fabrication of GaN-based devices. The surface roughness obtained is a root mean square (RMS) value calculated from AFM measurements. A study reported that the RMS roughness was lower under 100% N_2_ gas flow compared with an Ar–N mixed working gas flow because of the ion bombardment effect of the high-energy, high atomic mass argon ions if the other sputtering parameters were optimized. Therefore, modifying the total N_2_ flow rate to achieve a lighter or denser gas concentration was thought to improve the thin-film surface RMS value and grain density [47].

**Figure 6:**
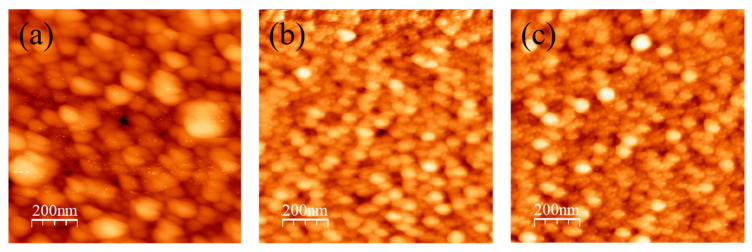
Two-dimensional AFM images of AlN thin films deposited using RF sputtering with N_2_ flow rates of (**a**) 20, (**b**) 30, and (**c**) 40 sccm.

Figure 6 presents AFM images of AlN thin films deposited using RF sputtering at N_2_ flow rates of 20, 30, and 40 sccm. The two-dimensional (2D) AFM images revealed a dense thin film. Some surface areas of the AlN film contain elongated grains; the elongated grains are the most and least prevalent in the 20 and 40 sccm samples. The increase in the N flow rate resulted in denser nitrogen concentrations, decreasing the crystal size because gas scattering reduces the kinetic energy of the nitrogen atoms, reducing the deposition rate and resulting in a lower RMS value. Using a more reactive gas for sputtering may lead to the formation of a denser structure with fewer voids in the grain boundaries [48]. The RMS value decreased from 13.17 nm to 2.57 nm as the N_2_ flow rate increased from 20 to 40 sccm. Thus, denser (and thus superior) AlN thin films with a lower RMS value and more uniform grain size can be obtained by increasing the N_2_ flow rate [49].

### 3.4. Hardness Measurement

The mechanical properties of the AlN thin film were studied through nanoindentation. AlN thin films have strong chemical bonds and are thus highly stable and resistant to degradation, even under harsh conditions. However, a better understanding of the mechanical characteristics of AlN thin films in the nanoscale regime is required for various device applications [50].

Material hardness is linked to interatomic bond lengths and the amount of covalent bonding. Furthermore, the bond strength depends on stoichiometry, the vacancy concentration, and impurities in the lattice structure. The microstructural features of the grain size in metal and alloy films affect the hardness of the material in accordance with the Hall–Petch relation. This hardness relation is also valid for polycrystalline materials [43]. Nevertheless, hardness depends not only on grain size but also on texture, porosity, and residual stress, which are influenced by thin film deposition conditions. The dislocation mobility for nitrides grown at 1000 °C is low, so it is essential to note that the grain boundaries play an important role in determining the hardness of AlN thin films [51].

**Figure 7:**
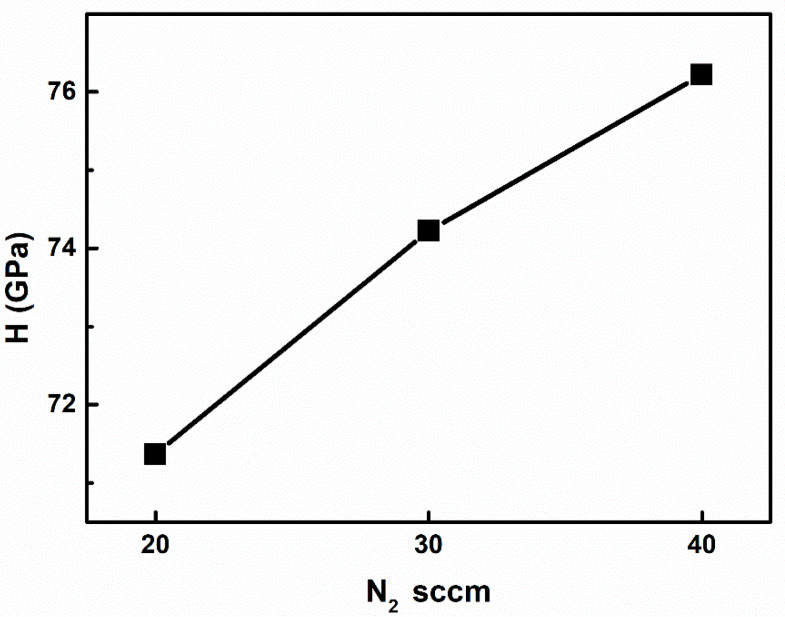
Hardness measurement plot of AlN thin films by varied N_2_ flow rate from 20 to 40 sccm.

Figure 7 reveals that hardness increases for higher values of the N_2_ ratio ranging from 71.67 to 76.21 GPa. The obtained hardness values are somewhat higher than those reported in the literature [52]. Al–N bonding increases as the N concentration in the sample increases, increasing the hardness of the sample. If a gas mixture is used for AlN thin-film formation, the hardness on the sample is more evenly dispersed throughout the sample (without significant deviation); the hardness can be observed from the XRD results revealing evenly grown AlN (002). The reported studies have also reported that the effect of modified gas flow ratio has a direct impact on the thin-film hardness [53,54].

### 3.5. XRD and Photoluminescence Spectroscopy for Si/GaN and Si/AlN/GaN

**Figure 8:**
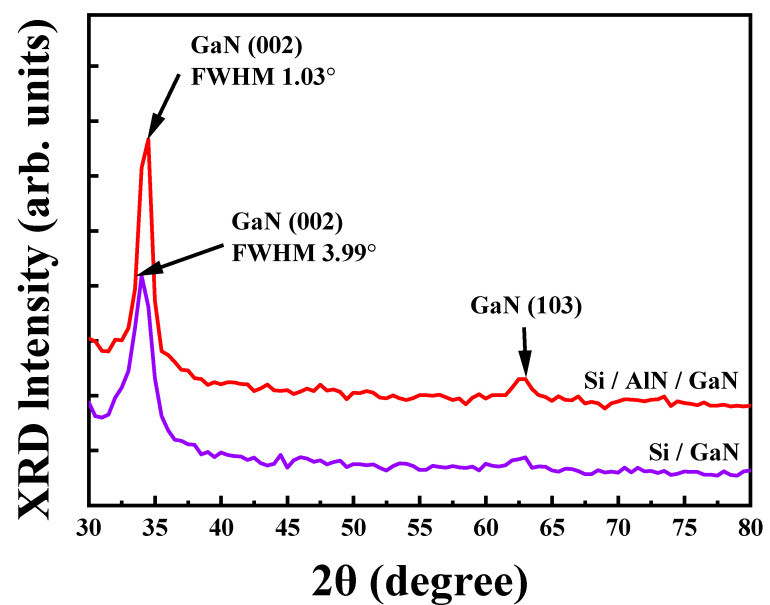
XRD patterns for Si/GaN and Si/AlN/GaN thin films.

AlN thin film produced with optimized sputtering parameters was used as the buffer layer for subsequent sputtering growth of GaN thin films on Si substrate; this Si/AlN/GaN thin film was compared with GaN sputtered directly on Si (Si/GaN). Figure 8 displays the XRD measurements for Si/GaN and Si/AlN/GaN and reveals GaN (002) and (103) phase orientations at 34° and 63°, respectively, for both samples. The obtained peaks formed in the hexagonal wurtzite phase and can be verified using the JCPDS card No. 01-089-7522. The intense and sharp peak at 34° indicates that the GaN crystals formed along the *c*-axis, confirming their (002) orientation [23]. A narrower GaN (002) peak, as indicated by a lower FWHM, indicates superior grain formation. The FWHMs for Si/GaN and Si/AlN/GaN were 3.99° and 1.03°, respectively. Thus, using AlN as a buffer layer improves GaN crystal formation.

**Figure 9:**
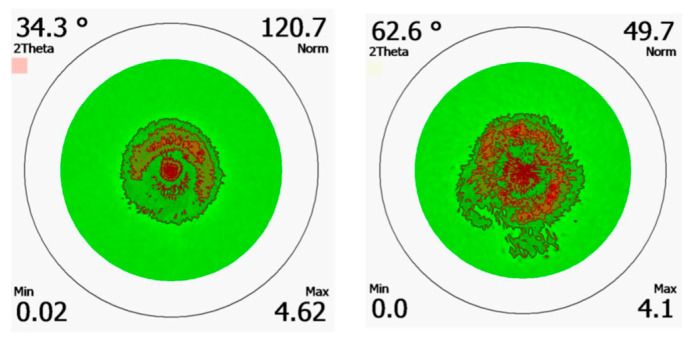
Pole figures for (002) and (103) Si/AlN/GaN stacked thin film structure.

XRD pole figures for the Si/AlN/GaN thin film structure are shown in Figure 9. The values corresponding to the GaN (002) and (103) at 34.3° and 62.6°, respectively, were used for the analysis, with the values of phi (Ψ) 0–360° and chi (χ) 0–75° for the measurement. The randomly oriented patterns from the pole figures for both GaN (002) and (103) suggest that the films are of polycrystalline nature. Since distinctive symmetric spots suggest the formation of crystalline orientation, further optimizations are needed on the stacking structure to improve the crystalline quality of the films [55,56].

**Figure 10:**
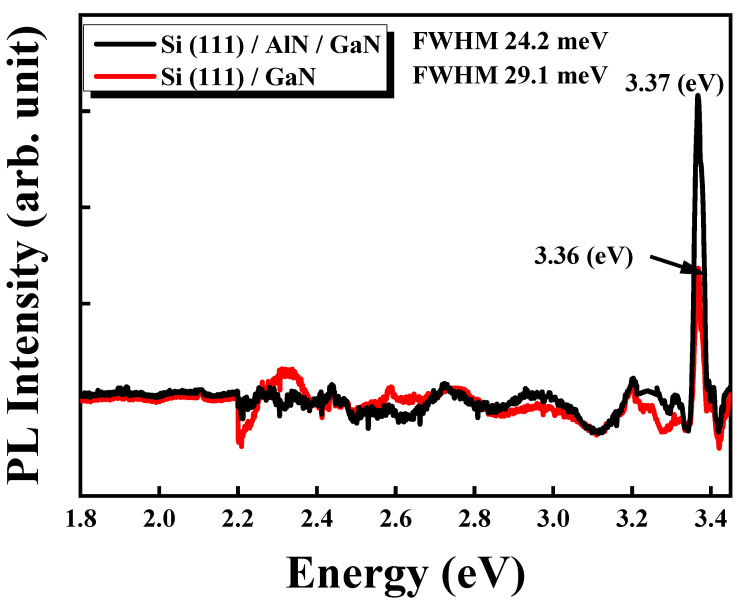
Photoluminescence spectra for Si/GaN and Si/AlN/GaN.

Figure 10 presents the PL spectra of Si/GaN and Si/AlN/GaN thin-film structures obtained with a He–Cd laser at a wavelength of 325 nm, excitation power of 30 mW, and measurement temperature of 10 K. In the measurement, a 0.5-meter monochromator (iHR 550) was used to measure the signal using a cooled charge-coupled device. PL spectroscopy was used to investigate the energy bands, impurities, energy transitions, crystal quality, and defect types of the GaN samples. The near-band-edge emission (NBE; 365 nm) of the GaN thin films deposited through sputtering was investigated; the near-band-edge emission is critical for developing high-quality GaN films for different applications. The main peaks for Si/AlN/GaN and Si/GaN samples were observed at 3.37 and 3.36 eV, respectively. The peak at 3.3 eV generally corresponds to NBE in GaN thin films, which crystallize with a hexagonal structure. It can also be ascribed to the recombination of excitons bound to neutral donors [57]. The PL FWHM for Si/GaN was 29.1 meV, and that for Si/AlN/GaN was 24.2 meV. Thus, the Si/AlN/GaN sample has superior PL FWHM and intensity and using an AlN film grown with optimized sputtering conditions as a buffer layer can promote crystal growth and quality of subsequently sputtered GaN thin films [58,59]. The results thus reveal that the NBE luminescence properties of the GaN thin films are appreciable.

Several researchers working with MBE and MOCVD have achieved superior PL characteristics for GaN thin films on AlN/Si substrate, but the use of sputtering to grow GaN films on AlN has received less attention. Typically, AlN is sputtered, and GaN is deposited using MOCVD, but the entire stack is not sputtered continually without breaking the vacuum. The process of not breaking the vacuum for the deposition of such thin films can reduce the cost and simplify the process. Thus, the optimum growth conditions for *c*-axis (002) AlN thin films and their potential as a buffer layer for hexagonal GaN thin films grown by subsequent RF sputtering have excellent potential for applications.

### 3.6. Secondary Ion Mass Spectrometry Measurement

**Figure 11:**
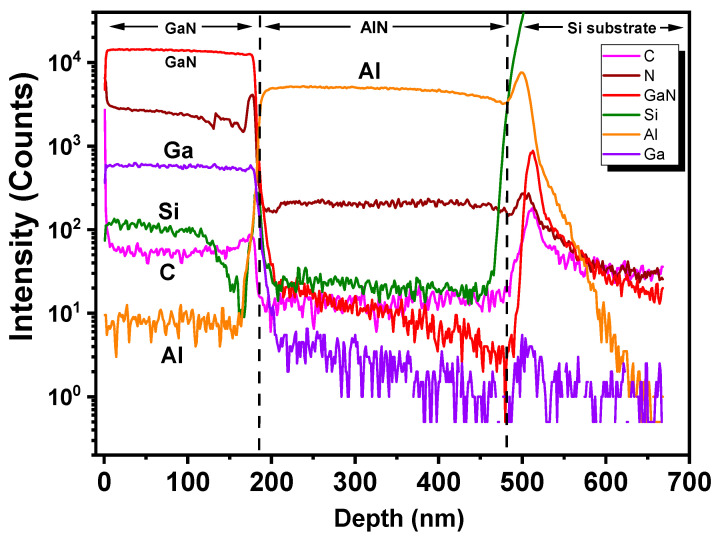
SIMS depth profile for the Si/AlN/GaN thin film structure.

The SIMS depth profile with surface etching for the Si/AlN/GaN thin film structure can be seen in Figure 11, indicating the sputtering thin-film growth of the GaN layer on the Si substrate with the AlN buffer layer. The presence of C can be attributed to atmospheric contamination, as also seen from our XPS results, suggesting unintentional impurity doping in the GaN layer and accounting for the carrier transition of 3.37 eV as observed in the PL results.

### 3.7. Hall-Effect Measurements

The Si/AlN/GaN thin-film structures were studied for their electrical properties using Hall-effect measurement. The nature of the sample was found to be of n-type with the carrier concentration of 1.27 × 10^15^ cm^−3^. The mobility was around 196.8 cm^2^ V^−1^cm^−1^. Resistivity was 24.9 Ω cm. The n-type nature of GaN thin films can be attributed to the Ga interstitials [60].

## 4. Conclusions

In this study, AlN thin films were deposited using RF sputtering on Si substrates. First, AlN films were deposited with a power of 300, 400, and 500 W. Next, the nitrogen gas ratio was varied at 50%, 75%, or 100% to study the effects of the film formation. Finally, the deposition temperature varied from 400 to 800 °C. Optimizing the parameters revealed that (002) AlN was best formed at 500 W, 600 °C, and with 100% N. The N_2_ flow rate was then varied from 20 to 40 sccm. The XRD results for these samples revealed that the sample with 40 sccm of N_2_ had high AlN (002) crystal quality. XPS surface level spectra were used to identify Al, N, O, and C peaks. The compositional at% of the AlN thin-film surface was calculated to be Al, 27.6%; N, 22.6%; and O, 46.4% at a N_2_ flow rate of 40 sccm. AFM 2D images revealed highly dense grain formation, and the surface roughness decreased from 13.17 nm to 2.57 nm as the N_2_ flow rate increased from 20 to 40 sccm. Hardness measurements were performed on the AlN samples, and the hardness of the AlN sample that was deposited using the N_2_ flow rate of 40 sccm was 76.21 GPa. Moreover, Si/AlN/GaN stack and Si/GaN thin-film structures were deposited using RF sputtering, and the XRD results indicated the formation of GaN (002) in the stack and directly on the Si. The PL spectra revealed an NBE emission of 3.37 eV for the GaN/AlN/Si film, and its PL FWHM was 24.2 meV. The results indicate that the RF-sputtered AlN thin films grown at 500 W, 100% N_2_ flow ratio, 600 ℃, and 40 sccm N_2_ flow are suitable for use as a buffer layer for the growth of a subsequent GaN layer for use in novel wide bandgap optoelectronic devices.

## Figures and Tables

**Table 1:** Various atomic percentages (at%) of elements identified in AlN thin films at the surface and at depths of 50 and 100 nm.

Sample	Surface			50 nm (Depth)			100 nm (Depth)		
	Al at%	N at%	O at%	Al at%	N at%	O at%	Al at%	N at%	O at%
**N 20**	15.1	10.1	72.4	24.4	40.5	25.1	25	40.4	24.6
**N 30**	21.1	12.6	62.3	26.9	43.8	20.5	26.8	44.5	21.7
**N 40**	27.6	22.6	46.4	31	50.5	15.6	32	52	12
